# Psychological characteristics of men with arterial hypertension according to the MMPI test

**DOI:** 10.1192/j.eurpsy.2022.1006

**Published:** 2022-09-01

**Authors:** N. Chernus, A. Sivkov, R. Gorenkov, S. Sivkov, A. Serdakova, A. Zolotovickaja, T. Savina

**Affiliations:** 1 the I.M. Sechenov First Moscow State Medical University: Moscow, Russia, The Outpatient Care Department, Moscow, Russian Federation; 2 the I.M. Sechenov First Moscow State Medical University: Moscow, Russia, The Outpatient Care Department, москва, Russian Federation

**Keywords:** hypertensive men, anxiety, frustration

## Abstract

**Introduction:**

The term hypertension means super-stress - psychoemotional. It is generally accepted that this condition is more inherent in men.

**Objectives:**

Study psychological features of arterial hypertension patients depending on severity.

**Methods:**

Examined 102 men, ages 31 to 62; the average age was 46.4 ± 0.32 years. Of these, with stage I AG 46 patients (1 group), with stage I AG 45 (2 group), with stage III AG 11 (3 group). Psychological status of patients was examined using the MMPI test.

**Results:**

The averaged personality profile of patients showed that a profile exceeding 80 T points is typical for all comparison groups: 82,6%, 73,3%, 81,8%, in groups 1, 2 and 3, respectively. The first profile type in frequency was the profile with a leading peak on the first scale in combination with a moderate rise on the right scales: seventh-eighth: 63.7% of the total number of surveyed. This profile reflected mainly depressive tendencies. The second most frequent profile reflected alarming trends: an increase on the 2nd scale with the main peak at 7. Persons with depressive manifestations were characterized by a focus on compliance with the normative criteria of the social environment, with anxious manifestations, personal characteristics were manifested by hostility, irritability, chronic social maladaptation

**Conclusions:**

Thus, the psychoemotional conditions identified are characterized by frustration, high levels of anxiety, interpersonal disorders, reduced performance, which may be considered mental health disorders

**Disclosure:**

No significant relationships.

